# A butanolic fraction from the standardized stem extract of *Cassia occidentalis* L delivered by a self-emulsifying drug delivery system protects rats from glucocorticoid-induced osteopenia and muscle atrophy

**DOI:** 10.1038/s41598-019-56853-6

**Published:** 2020-01-13

**Authors:** Subhashis Pal, Naresh Mittapelly, Athar Husain, Sapana Kushwaha, Sourav Chattopadhyay, Padam Kumar, Eppalapally Ramakrishna, Sudhir Kumar, Rakesh Maurya, Sabyasachi Sanyal, Jiaur R. Gayen, Prabhat R. Mishra, Naibedya Chattopadhyay

**Affiliations:** 1 Division of Endocrinology and Center for Research in Anabolic Skeletal Target in Health and Illness (ASTHI), CSIR-Central Drug Research Institute, Council of Scientific and Industrial Research, Lucknow, 226031 India; 2 Division of Pharmaceutics, CSIR-CDRI, Lucknow, 226031 India; 3 Division of Pharmacokinetics, CSIR-CDRI, Lucknow, 226031 India; 4 Division of Biochemistry, CSIR-CDRI, Lucknow, 226031 India; 5 Division of Medicinal & Process Chemistry, CSIR-CDRI, Lucknow, 226031 India; 6 AcSIR, CSIR-Central Drug Research Institute Campus, Lucknow, 226031 India

**Keywords:** Pharmaceutics, Bone

## Abstract

We recently reported that a butanol soluble fraction from the stem of *Cassia occidentalis* (CSE-Bu) consisting of osteogenic compounds mitigated methylprednisone (MP)-induced osteopenia in rats, albeit failed to afford complete protection thus leaving a substantial scope for further improvement. To this aim, we prepared an oral formulation that was a lipid-based self-nano emulsifying drug delivery system (CSE-BuF). The globule size of CSE-BuF was in the range of 100–180 nm of diluted emulsion and the zeta potential was −28 mV. CSE-BuF enhanced the circulating levels of five osteogenic compounds compared to CSE-Bu. CSE-BuF (50 mg/kg) promoted bone regeneration at the osteotomy site and completely prevented MP-induced loss of bone mass and strength by concomitant osteogenic and anti-resorptive mechanisms. The MP-induced downregulations of miR29a (the positive regulator of the osteoblast transcription factor, Runx2) and miR17 and miR20a (the negative regulators of the osteoclastogenic cytokine RANKL) in bone was prevented by CSE-BuF. In addition, CSE-BuF protected rats from the MP-induced sarcopenia and/or muscle atrophy by downregulating the skeletal muscle atrogenes, adverse changes in body weight and composition. CSE-BuF did not impact the anti-inflammatory effect of MP. Our preclinical study established CSE-BuF as a prophylactic agent against MP-induced osteopenia and muscle atrophy.

## Introduction

Worldwide nearly 60% of patients of rheumatoid arthritis (RA) are treated with glucocorticoids (GC)^1^. Prednisone/methylprednisolone, the most frequently used GC is given in combination with disease modifying anti-rheumatic drugs (DMARDs) to provide better clinical benefit. However, long-term use of GC has several metabolic adverse effects and the most prominent among those being osteoporosis and sarcopenia^2,3^. The clinically used drugs for GC-induced osteoporosis (GIO) are anti-resorptives (bisphosphonates and denosumab) whereas the disease is primarily an effect of impairment of osteoblast function^4^. Although the only osteoanabolic drug, human parathyroid hormone 1–34 (teriparatide) has shown therapeutic efficacy greater than bisphosphonates in GIO^5–10^ this drug has not received approval for use in this indication from the U.S. Food & Drug Administration (FDA), thus leaving a scope for discovery of new bone forming agent. Long-term GC treatment also causes sarcopenia^11^. Skeletal muscle provides a positive signal to bones via biomechanical loading and sarcopenia for which there is no drug further impairs bone health.

*Cassia occidentalis* L. (belongs to *Caesalpiniaceae* family) is an annual plant that is abundantly distributed in wide areas of South Asia and South America. Traditional uses of leaf and stem of *C. occidentalis* for the treatment of fracture and ailments of bone are known since the late nineteenth century in Puttur, a census town of Chittoor district of Andhra Pradesh, a Southern state of India^12,13^. We have recently shown that a butanolic fraction made from the ethanolic extract of *C. occidentalis*’s stem promotes bone regeneration at the fracture site and protected against methylprednisone (MP)-induced loss of bone mass and strength in rats^14^. In this regard, the butanolic fraction (CSE-Bu) was found to be more potent than the ethanolic extract. Although CSE-Bu provided significant protection against the MP-induced bone loss, however, it was not complete, thus leaving a scope for improving its efficacy.

Phytochemicals are known for their poor oral bioavailability and to achieve *in vivo* efficacy of a given agent, higher doses are required which in turn reduces its therapeutic window^15^. Self-nano emulsifying drug delivery system (SEDDS) is an efficient approach for enhancing intestinal absorption of hydrophobic compounds that are present in CSE-Bu, leading to their improved bioavailability and more consistent temporal profile of their absorption. CSE-Bu contains six osteogenic compounds out of which isovitexin had the best osteogenic effect *in vitro*^14^.

Here, we developed a lipid-based SEDDS of CSE-Bu (CSE-Bu formulation denoted henceforth as CSE-BuF) to enhance absorption and consequently bioavailability of the osteogenic compounds present in CSE-Bu. We then studied the pharmacokinetics and pharmacodynamics of CSE-BuF. Furthermore, we studied the effect of CSE-BuF on the anti-inflammatory effect of MP. Next, we used a rat femur osteotomy model to assess bone regeneration and an MP-induced osteopenia model to assess the efficacy of CSE-BuF, and studied its action mechanism in bone. Since MP is also known to cause sarcopenia which in turn contributes to bone loss due to reduced mechanical loading, we thus studied the effect of CSE-BuF on the impact of MP on skeletal muscle.

## Results

### Characterization of CSE-BuF and its effect on the oral bioavailability of osteogenic compounds

**Figure 1:**
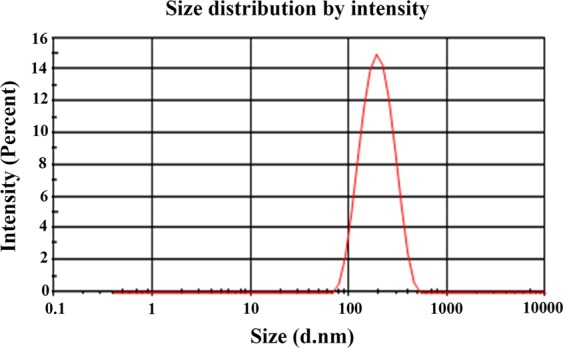
Globule size and size distribution of SEDDS after dilution. The average globule size and PDI of formulation after dilution 1:100 in distilled water was found to be was 166.7 d.nm and 0.186 respectively.

The globule size of CSE-BuF was found to be in the range of 100–170 nm of diluted emulsion and the zeta potential was −28 mV (Fig. 1).

Previously we isolated five osteogenic compounds including emodin, luteolin, 3′,4′,7-trihydroxyflavone (THF), apigenin and isovitexin from CSE-Bu based on *in vitro* assays^14^. Among these compounds, emodin and luteolin could be measured in plasma after single oral dosing of CSE-Bu whereas apigenin, isovitexin, and THF were not detectable (Table 1). However, single oral dosing of CSE-BuF, resulted in increases in plasma emodin and luteolin levels over the CSE-Bu treatment. Compared to CSE-Bu, the relative bioavailability of emodin and luteolin was increased respectively by 279% and 36% by CSE-BuF treatment. In contrast to CSE-Bu, apigenin, isovitexin and THP levels were detectable and measured in plasma of rats given single oral dosing of CSE-BuF (Table 1).

**Table 1:** Pharmacokinetics of osteogenic compounds in CSE-Bu and CSE-BuF.

	CSE-Bu at 250 mg/kg orally		CSE-BuF at 250 mg/kg orally		%Fr
	Cmax (ng/mL)	AUC (hr*ng/mL)	Cmax (ng/mL)	AUC (hr*ng/mL)	
Apigenin	ND	ND	3.98 ± 3.03	2.20 ± 1.15	—
Apigenin 6-C-glucoside Or Isovitexin	ND	ND	7.74 ± 4.29	17.90 ± 10.36	—
3′,4′,7-trihydroxyflavone (THF)	ND	ND	200.52 ± 107.36	764.11 ± 381.83	—
Emodin	12.35 ± 3.80	69.30 ± 33.24	29.25 ± 12.98	239.13 ± 31.07	379.68 ± 87.63
Luteolin	78.68 ± 34.97	259.17 ± 144.44	343.32 ± 136.61	456.33 ± 131.28	136.94 ± 17.14

Fr: Relative bioavailability, ND: Not detected, values represents Mean ± SD.

### CSE-BuF enhanced the bone regenerative effect

**Figure 2:**
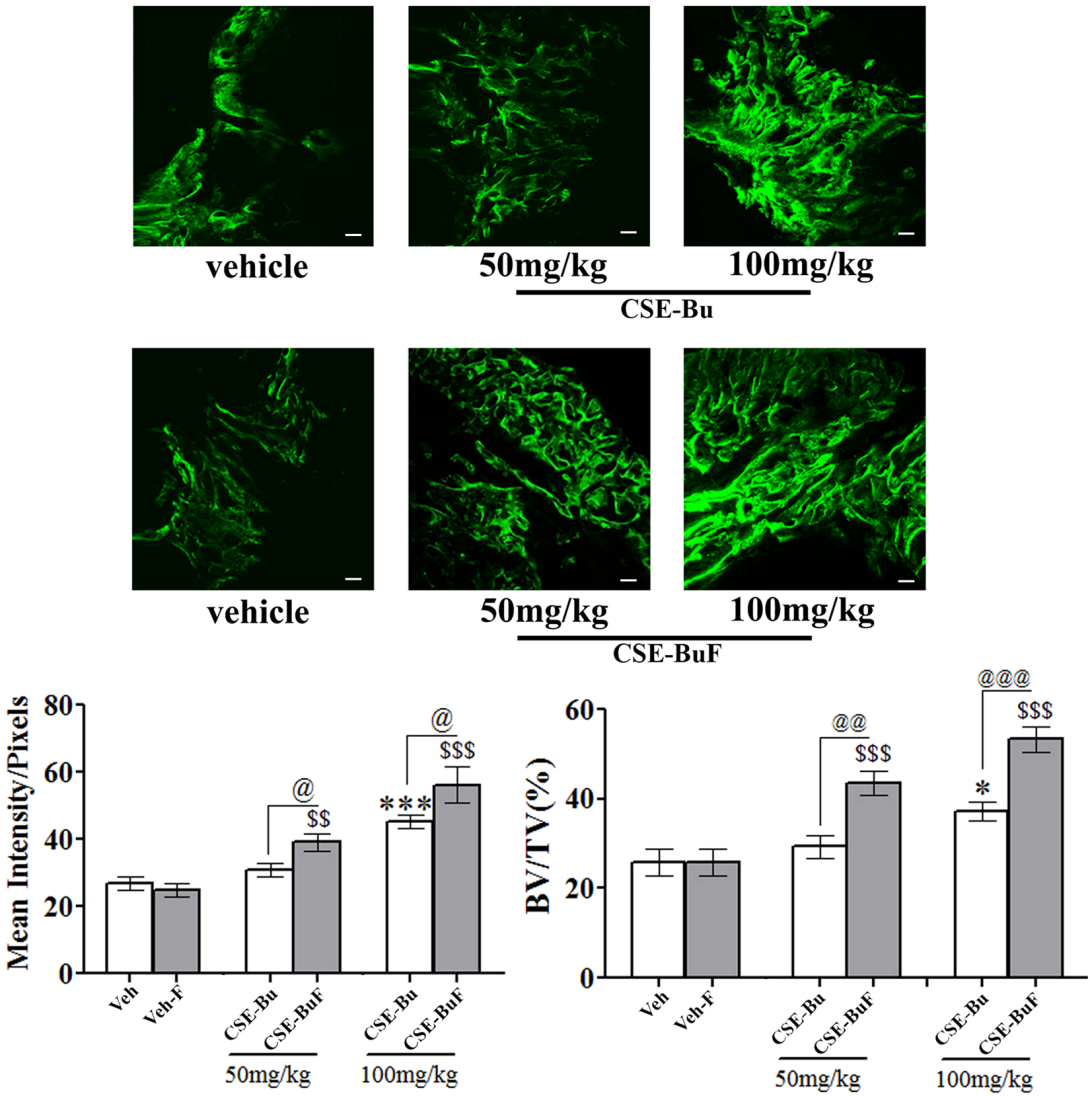
CSE-BuF enhanced the bone regenerative effect of the butanolic fraction of the stem extract of *Cassia occidentalis* (CSE-Bu). Representative micrographs of calcein labeled callus from various groups (upper panel) and quantified data of calcein labeling and µCT analysis of callus at fracture site (lower panel). Scale bar, 50 µm. Water was used as vehicle (veh) for CSE-Bu group and blank SEDDS without extract as vehicle (veh-F). Data are expressed as mean ± SEM (n = 8–12/group); *p < 0.05 and ***p < 0.001 versus veh; ^$$^*p* < 0.01 and ^$$$^*p* < 0.001 versus veh-F; ^@^*p* < 0.05, ^@@^*p* < 0.01 and ^@@@^*p* < 0.001 were used to compare between the formulated and unformulated groups.

Bone regeneration, assessed by calcein labeling at the femur osteotomy site, was compared between CSE-BuF and CSE-Bu. Compared to the vehicle (water), the formulation of the vehicle (blank SEDDS without extract) had no effect on calcein labeling. At 50 mg/kg dose, CSE-Bu had no effect but CSE-BuF significantly increased mean calcein intensity. Micro CT (μCT) assessment showed increased callus bone volume (BV/TV) in the CSE-BuF compared to the CSE-Bu group (Fig. 2).

### CSE-BuF completely prevented the osteopenic effect of MP

Since CSE-BuF at 50 mg/kg dose stimulated significant bone regeneration at the osteotomy site, we tested whether at this dose it could prevent the osteopenic impact of MP. In the MP group, a significant decrease in body weight (26%) appeared to be contributed by the decrease of both lean (11%) and fat mass (40%) compared to control. CSE-BuF prevented the MP-induced loss of body weight by maintaining lean- and fat mass to the control levels (Table 2).

**Table 2:** Body weight and composition in various groups.

	Vehicle (control)	MP	MP + CSE-BuF (50 mg/kg)
Body weight (gm)	321.93 ± 8.36	239.86 ± 5.71^***^	308.02 ± 8.47
Lean Mass (%)	81.02 ± 0.83	71.96 ± 2.43^**^	81.23 ± 1.07
Fat Mass (%)	10.23 ± 0.55	6.12 ± 0.58^***^	9.36 ± 0.69

Data are expressed as mean ± SEM (n = 10/group); ^**^P < 0.01 and ^***^P < 0.001 versus vehicle.

**Figure 3:**
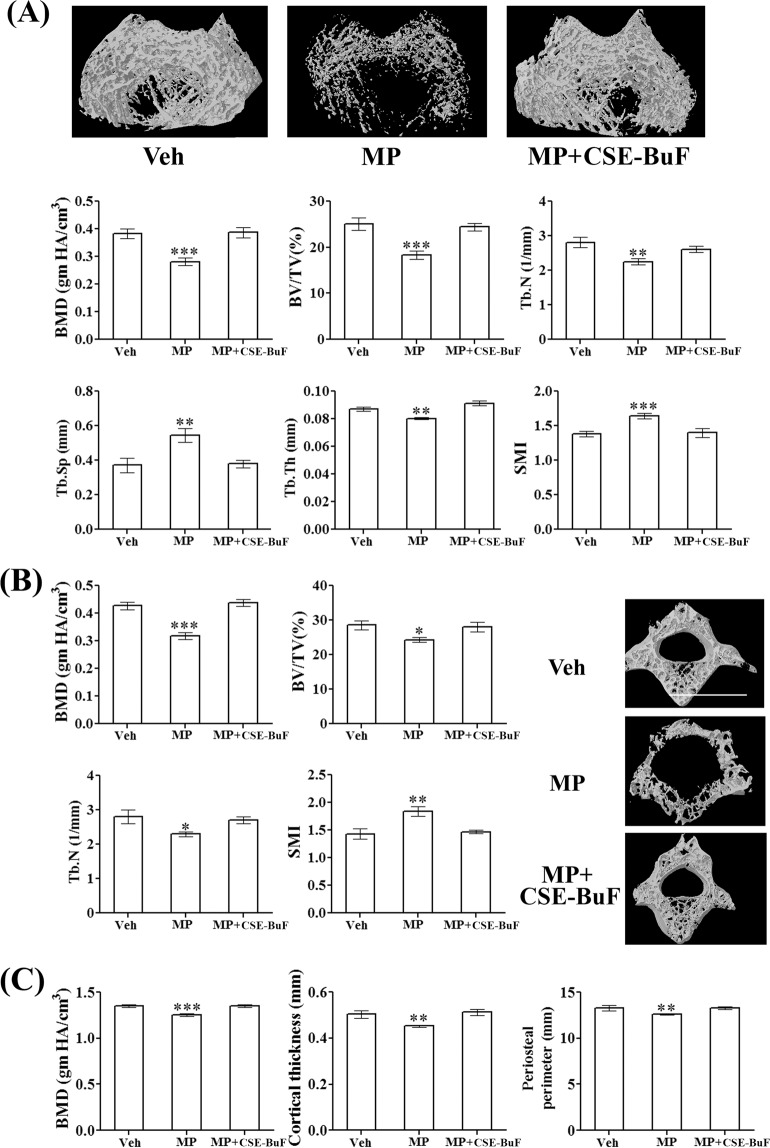
CSE-BuF prevented the osteopenic effect of MP. (**A**) Upper panel showing 3-D representative images of different groups. Volumetric BMD (vBMD) and various trabecular parameters including BV/TV, Tb.N, Tb.Th, Tb.Sp and SMI of femur metaphysis are shown in the lower panels. (**B**) Left panel showing various trabecular parameters of L5 vertebra and right panel showing the 3-D representative images of indicated groups. (**C**) Assessment of cortical parameters at femur diaphysis was done by vBMD, cortical thickness and periosteal perimeter. Data are expressed as mean ± SEM (n = 8–10/group); *P < 0.05, **P < 0.01 and ***P < 0.001 compared to vehicle (Veh).

CSE-BuF completely protected femur metaphysis (trabecular bones), femur diaphysis (cortical bone) and L5 vertebra (trabecular bone) from the MP-induced loss (Fig. 3A–C). In femur metaphysis and L5, bone mineral density (BMD), bone volume (BV/TV%) and microarchitectural parameters [trabecular number (Tb.N), trabecular thickness (Tb.Th), and trabecular separation (Tb.Sp)] were comparable between the control and CSE-BuF groups (Fig. 3A,B). Structure model index (SMI), an indicator of trabecular bone geometry was also comparable between these two groups (Fig. 3A,B). In addition, CSE-BuF completely prevented the MP-induced loss of BMD, cortical thickness and periosteal perimeter of femur diaphysis (Fig. 3C).

**Figure 4:**
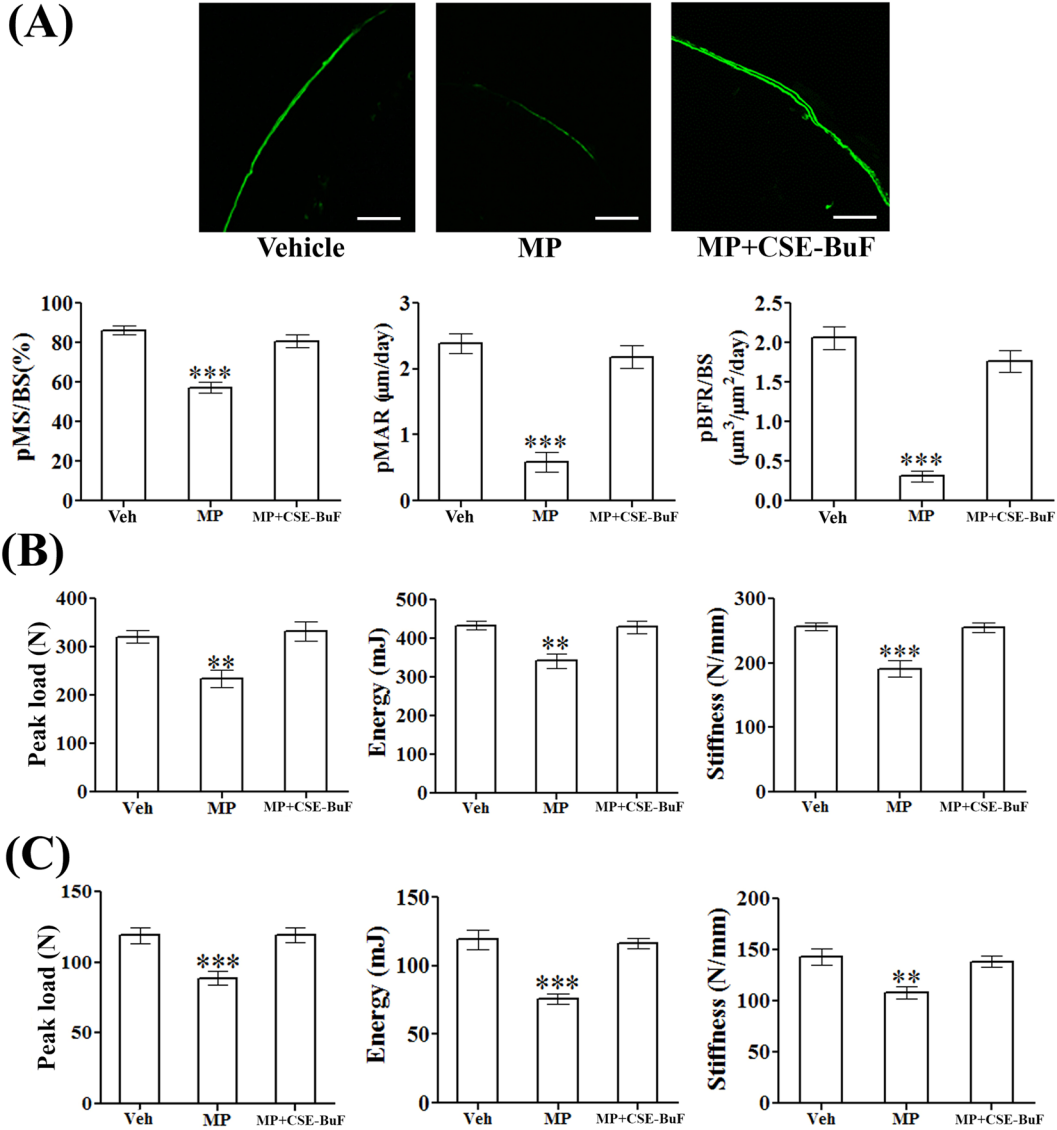
CSE-BuF promoted bone formation and bone strength in MP-treated rats. (**A**) Upper panel showing representative calcein labelling (20X) at femur diaphysis in the indicated groups and lower panel showing the quantified data including periosteal (p)-mineralizing surface/bone surface (p-MS/BS), mineral apposition rate (pMAR), and bone formation rate/bone surface (pBFR/BS). Scale bar, 50 µm. (**B**) L5 compression test and (**C**) femur 3-point bending of indicated groups are shown. Data are expressed as mean ± SEM (n = 6/group) **P < 0.01 and ***P < 0.001 compared to vehicle (Veh).

We performed calcein double labeling analysis at femur diaphysis to measure bone formation rate. Representative micrograph showed intense and continuous calcein labeling of femur diaphysis that was interspersed by closely apposed double labeling in the control and MP + CSE-BuF groups in contrast to the faint and broken single labeling observed in the MP group (Fig. 4A, upper panel). Calculation of dynamic parameters of the bone formation based on double calcein labeling experiment showed significant decreases in the mineralizing surface (pMS/BS), mineral apposition rate (pMAR) and bone formation rate (pBFR/BS) in the MP group compared to control (Veh). These parameters were comparable between the control and CSE-BuF groups (Fig. 4A, lower panel).

Since the loss of bone mass and microarchitecture cause loss of bone strength, we studied the compressive strength of L5 vertebra and 3-point bending strength of femur diaphysis. MP treatment decreased all strength parameters (peak load, energy to failure and stiffness) in L5 vertebra and femur diaphysis, and CSE-BuF maintained all three parameters at both sites to the levels of control rats (Fig. 4B,C).

### CSE-BuF prevented reduced bone formation and increased resorption by MP without affecting its immune suppression effect

**Figure 5:**
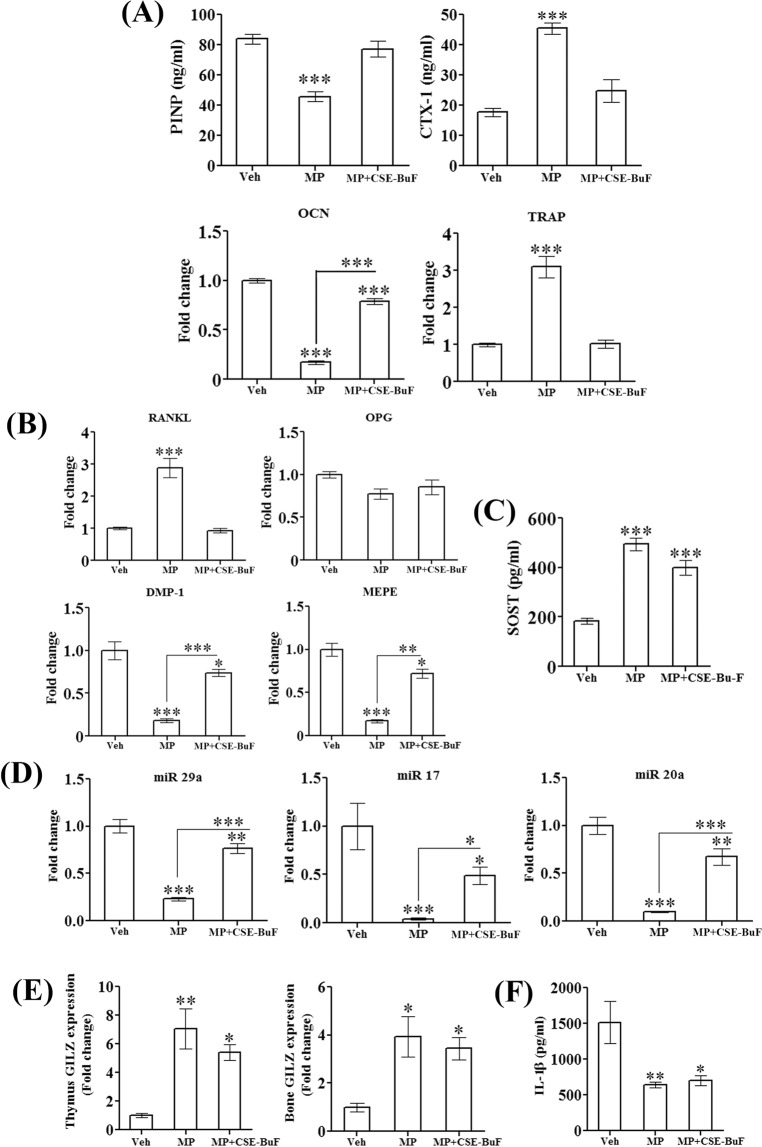
CSE-BuF promoted osteoblast activity and suppressed resorption without altering anti-inflammatory property of methylprednisolone. (**A**) Assessment of serum turnover markers (PINP and CTX-1), bone-specific osteogenic (OCN) and resorptive (TRAP) gene expression in the indicated groups. (**B**) Assessment of osteocyte markers (DMP-1, MEPE), RANKL and OPG in bones of indicated groups by qPCR. (**C**) Assessment of serum sclerostin (SOST) levels in the indicated groups by ELISA. (**D**) Assessment of expression of various corticosteroid-regulated miRNAs (miR 29a, miR17 and miR20a) involved in bone remodeling in the indicated groups. (**E**) Assessment of expression of GLIZ mRNA in thymus and proximal femur of indicated groups by qPCR. (**F**) Assessment of serum IL-1β levels in the indicated groups by ELISA. Data are expressed as mean ± SEM (n = 6/group) *P < 0.05, **P < 0.01 and ***P < 0.001 compared to vehicle (veh).

To study the mechanism of osteopotection by CSE-BuF, we first measured serum bone turnover markers. MP suppressed procollagen type-I N-terminal propeptide (PINP, bone formation marker) and increased cross-linked C-telopeptide of type-I collagen (CTX-I, bone resorption marker) compared to control (Veh) and CSE-BuF maintained both to the levels of the control (Fig. 5A). Consistent with reduced PINP, the mRNA level of osteocalcin (OCN), the osteoblast produced non-collagen matrix protein was significantly suppressed in the MP group compared with control and CSE-BuF treatment significantly increased it over the MP group (Fig. 5A). Furthermore, keeping with the increased serum resorption marker, we observed a robust increase in tartrate-resistant acid phosphatase (TRAP, osteoclast-specific enzyme) mRNA in bones of MP treated group over the control and CSE-BuF suppressed it to the control level (Fig. 5A).

Osteocytes are mature osteoblasts trapped in bone matrix and serve as a major regulator of bone remodeling by secreting Receptor activator of nuclear factor kappa-Β ligand (RANKL)^16^. Apoptotic osteocytes are robust producers of RANKL, the key osteoclastogenic cytokine^17,18^. GCs are known to induce osteocyte apoptosis^19^, and accordingly, we observed a significant decrease in the mRNA levels of osteocyte markers: dentin matrix protein 1(DMP-1), matrix extracellular phosphoglycoprotein (MEPE) and a robust increase in RANKL in bones of MP group compared to control, and CSE-BuF prevented the MP-induced changes (Fig. 5B). Sclerostin, primarily an osteocyte-produced protein that inhibits osteoblast function was significantly increased in the serum of MP treated rats compared to control, however, CSE-BuF had no effect compared with MP treated group (Fig. 5C).

To gain insight into the molecular mechanism of the action of CSE-BuF, we next measured different micro RNA (miRNA) levels in bones that are known to be altered by GCs. miRNA 29a is known to promote osteoblast activity via the regulation of Runx2 expression and MP suppresses its expression^20^. Both miRNA 17 and miRNA 20a inhibit RANKL expression by osteoblast and GCs suppress their expression^21,22^. Consistent with these reports, we observed significant decreases in the levels of miRNA 17, −20a and −29a in bones of MP treated rats compared to control, and CSE-BuF treatment significantly increased the levels of all three miRNAs compared to the MP group (Fig. 5D).

We next checked whether prevention of the osteopenic effect of MP by CSE-BuF involved an alteration in the anti-inflammatory effect of MP. Glucocorticoid-induced leucine zipper (GILZ) mediates the anti-inflammatory action of GCs in various organs, most importantly by regulating the thymic T cell activities^23^. MP treatment resulted in robust increase of GILZ mRNA in thymus and bone over the control, and CSE-BuF did not alter the effect of MP (Fig. 5E). Besides, the serum levels of the inflammatory cytokine IL-1β was suppressed by MP treatment and CSE-BuF had no effect on the MP-induced suppression (Fig. 5F).

### CSE-BuF prevented muscle atrophy caused by MP

**Figure 6:**
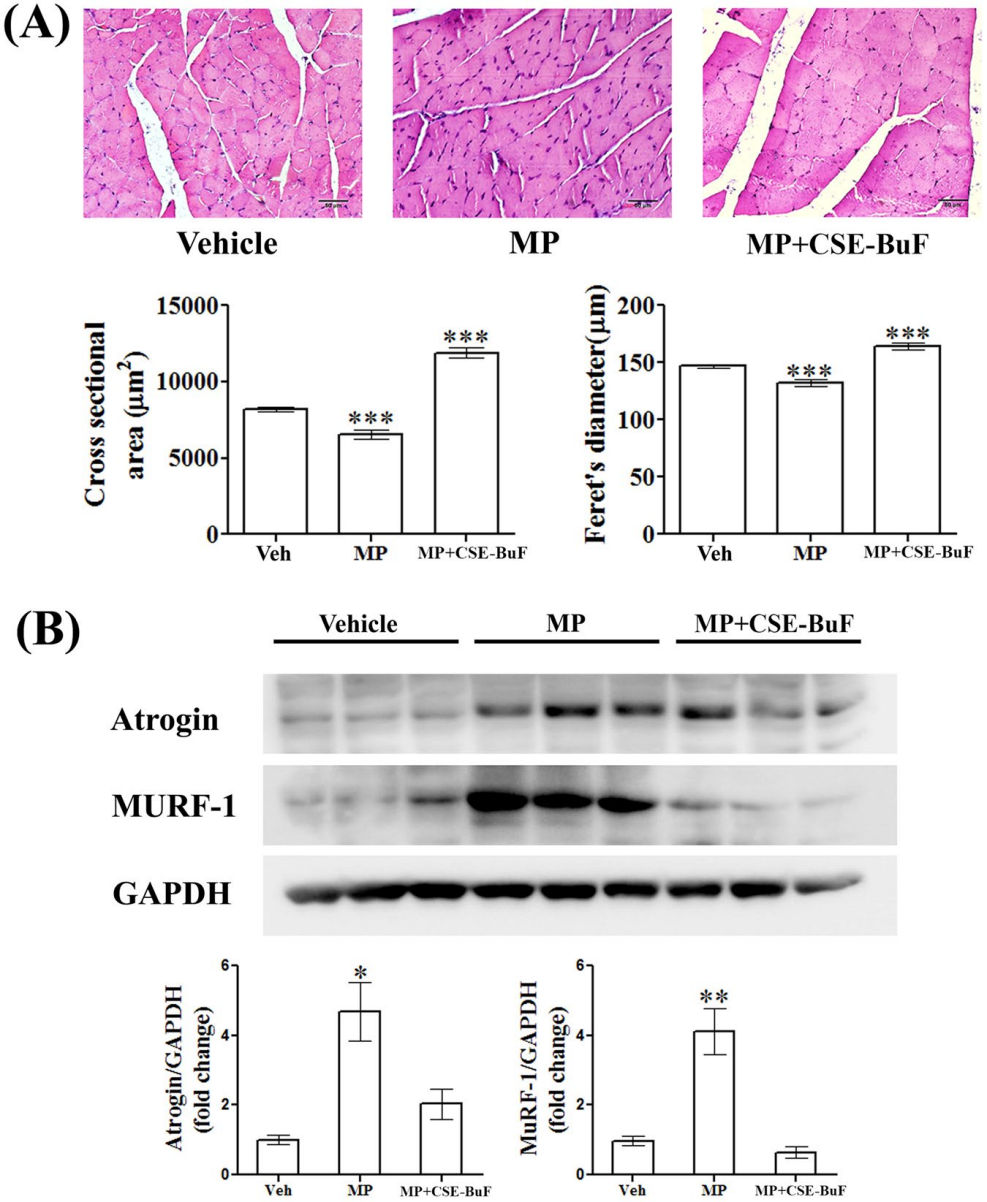
CSE-BuF prevented MP-induced muscle atrophy. (**A**) Upper panel showing representative H&E stained sections of gastrocnemius muscle (40X) and the lower panel showing quantified cross sectional area and feret’s diameter of muscle fibers in the indicated groups. Scale bar, 50 µm. (**B**) Western blot images and densitometric quantification of bands of atrogin-1 and MuRF-1 expression in gastrocnemius muscle from rats of the indicated groups are shown. Data are expressed as mean ± SEM (n = 6/group) *P < 0.05, **P < 0.01 and ***P < 0.001 compared to vehicle (veh).

MP-induced muscle atrophy was observed from the significantly reduced cross-sectional area and Feret’s diameter of gastrocnemius muscle compared to control and both parameters in CSE-BuF group were higher than the control (Fig. 6A). Furthermore, MP treatment increased the muscle catabolic proteins including atrogin and muscle RING-finger protein-1 (MuRF-1) levels, thus causing muscle atrophy, and CSE-BuF completely suppressed their levels to that of the control (Fig. 6B).

## Discussion

SEDDS is emerging as an effective carrier system for improving the bioavailability of poorly absorbed compounds present in plant extracts. Although most reports used pure phytochemicals, this approach has also been used to enhance the bioavailability of methoxyflavones of *Kaempferia parviflora* extract^24^, germacrone of dry rhizome of *Curcuma zedoaria* extract^25^, bilabolide and ginkgolide A and B of *Ginkgo biloba* extract^26^ and protopine and tetrahydropalmatine of Rhizoma *Corydalis decumbentis*^27^.

Our data show that CSE-BuF, a SEDDS-based formulation at 50 mg/kg dose promotes bone regeneration and completely prevents MP-induced osteo-sarcopenia in rats likely by robustly augmenting the bioavailability of five osteogenic compounds (apigenin, isovitexin, THP, emodin, and luteolin) reported by us based on *in vitro* assays^14^. Among these, *in vivo* bone conserving effect has been reported for apigenin, emodin, and luteolin^28–32^. Isovitexin also has *in vivo* osteogenic effect in mice (unpublished data, S. Pal and N. Chattopadhyay).

Consistent with the increased oral bioavailability of osteogenic compounds, we observed a more potent osteogenic effect of CSE-BuF (at 50 mg/kg) in both the models used here compared to our previously reported ethanolic extract of *C. occidentalis* stem (CSE, effective dose, 250 mg/kg) and CSE-Bu (effective dose 100 mg/kg)^14^. Despite higher doses, CSE or CSE-Bu failed to provide complete skeletal protection against MP-induced bone loss which CSE-BuF did^14^, which suggested that CSE-BuF was significantly better than the unformulated extract/fraction.

The skeletal effect of CSE-BuF involved both osteoanabolic and anti-resorptive mechanisms. Given that osteoanabolic therapy (teriparatide) has a better therapeutic effect in GIO than bisphosphonates^6–10^ the most commonly used anti-resorptive therapy, and since teriparatide is not an FDA approved drug for GIO, there is a need for osteoanabolic intervention for GIO, which could potentially be served by CSE-BuF. Furthermore, sarcopenia caused by MP in turn secondarily affects bone mass, and it was prevented by CSE-BuF. In addition, the adverse changes in body composition caused by MP displaying decreases in both lean and fat mass were prevented by CSE-BuF. Taken together, CSE-BuF provides complete protection against MP-induced changes in osteopenia and sarcopenia that secondarily impact bone health.

MP caused erosion of trabecular bones at femur metaphysis and lumbar vertebra, the clinically relevant sites for fracture incidence^33,34^. CSE-BuF completely protected both sites from the MP-induced osteopenic impact which was further translated to the maintenance of L5 compression strength, which suggested that CSE-BuF could protect compression fracture of trabecular bones by MP. With chronic MP treatment, trabecular bone loss is accompanied by cortical bone loss^33^. We observed significant decreases in femur diaphyseal (cortical bone) mass and cortical thickness leading to loss of bending strength by MP treatment, and CSE-BuF completely protected bones from these adverse effects. Cortical osteopenia by MP was confirmed by diminished surface referent bone formation indicated by decreased MS/BS (percentage of bone surface undergoing active formation), MAR (average rate of osteoblast activity at each bone multicellular unit) and BFR (bone formation rate) at femur diaphysis and CSE-BuF maintained these parameters to control levels. Although surface referent bone formation parameters have not been assessed in trabecular bones, it is reasonable to extrapolate the finding from the cortical bones to conclude that CSE-BuF stimulated osteoblast function (anabolic effect) to afford protection against MP-induced osteopenia at both skeletal sites. Moreover, because serum PINP is reduced in patients with GIO^35^, this could be monitored as a biological response marker for CSE-BuF in the clinical setting.

Osteocytes are matrix resident osteoblasts that critically regulate bone homeostasis and one of the major mechanisms by which MP induces osteopenia is by inducing osteocyte apoptosis^36^. A significant reversal of the strong suppressive effect of MP by CSE-BuF on the osteocyte genes (DMP-1 and MEPE) attests to the later’s osteoanabolic effect, however, it failed to suppress the MP-induced rise in serum sclerostin levels. Our finding of suppression of DMP-1 and MEPE by MP is different from a report showing no effect of prednisolone on the lumbar vertebra of mice^37^. This difference could be explained by the fact that the control mice were female C57BL/6 carrying Atg 7-f/f transgene whereas we used male SD rats and the glucocorticoid dose was ~60% lesser (2.1 mg/kg) than our dose (5 mg/kg). It thus appears from our study that reduction in osteocyte markers is associated with the increased production of sclerostin by MP and whereas CSE-BuF mitigates the effect of MP on the expression of the marker it does not affect the elevated levels of the osteoblast suppressing Wnt inhibitor, sclerostin. From our data, it appears that the osteoanabolic effect of CSE-BuF is not due to the suppression of sclerostin levels. Furthermore, glucocorticoids are known to induce osteocyte apoptosis^38,39^, and apoptotic osteocytes robustly produce the potent osteoclastic cytokine, RANKL^40^, and CSE-BuF completely blocked the mRNA levels of this cytokine induced by MP. As a result, CSE-BuF prevented enhanced bone resorption caused by MP which was evident from robustly increased serum CTX-1. At the molecular level, MP downregulated the RANKL inhibitory miRNA 17- and 20a, and Runx2 and β-catenin stabilizing miRNA 29a in bones and CSE-BuF prevented these changes. These data provide the cellular and molecular mechanisms underlying the protective effect of CSE-BuF on MP-induced bone loss.

Observations of a strong association between osteopenia with sarcopenia in several diseases and aging led to the identification of bone and skeletal muscle as tissues that are anatomically and functionally linked. Loss of muscle mass contributes to bone loss due to reduced mechanical loading of bones^41^. Chronic GC use is known to cause sarcopenia by stimulating muscle catabolic pathway^11^ and consistent to this finding, we observed decreased muscle mass due to reduced muscle size contributed by the upregulation of atrogin-1 and MurF-1, the proteins belonging to E3 ubiquitin ligase family.

Safety and toxicity issues are critical to the clinical translation of a therapeutic intervention. Ethanolic extract of *C. occidentalis* up to 2.5 g/kg had no toxicity in acute and sub-acute studies^42^. Although we have not carried out detailed toxicity studies with CSE-BuF, however, there were no mortality and weight loss due to CSE-BuF treatment. Rather, CSE-BuF completely protected against the MP-induced loss of body weight (both lean and fat mass) thus suggesting its safety. Moreover, the formulation excipients are “Generally Recognized as Safe” (GRAS) category as per the U.S. FDA and unlikely to have toxicity.

**Figure 7:**
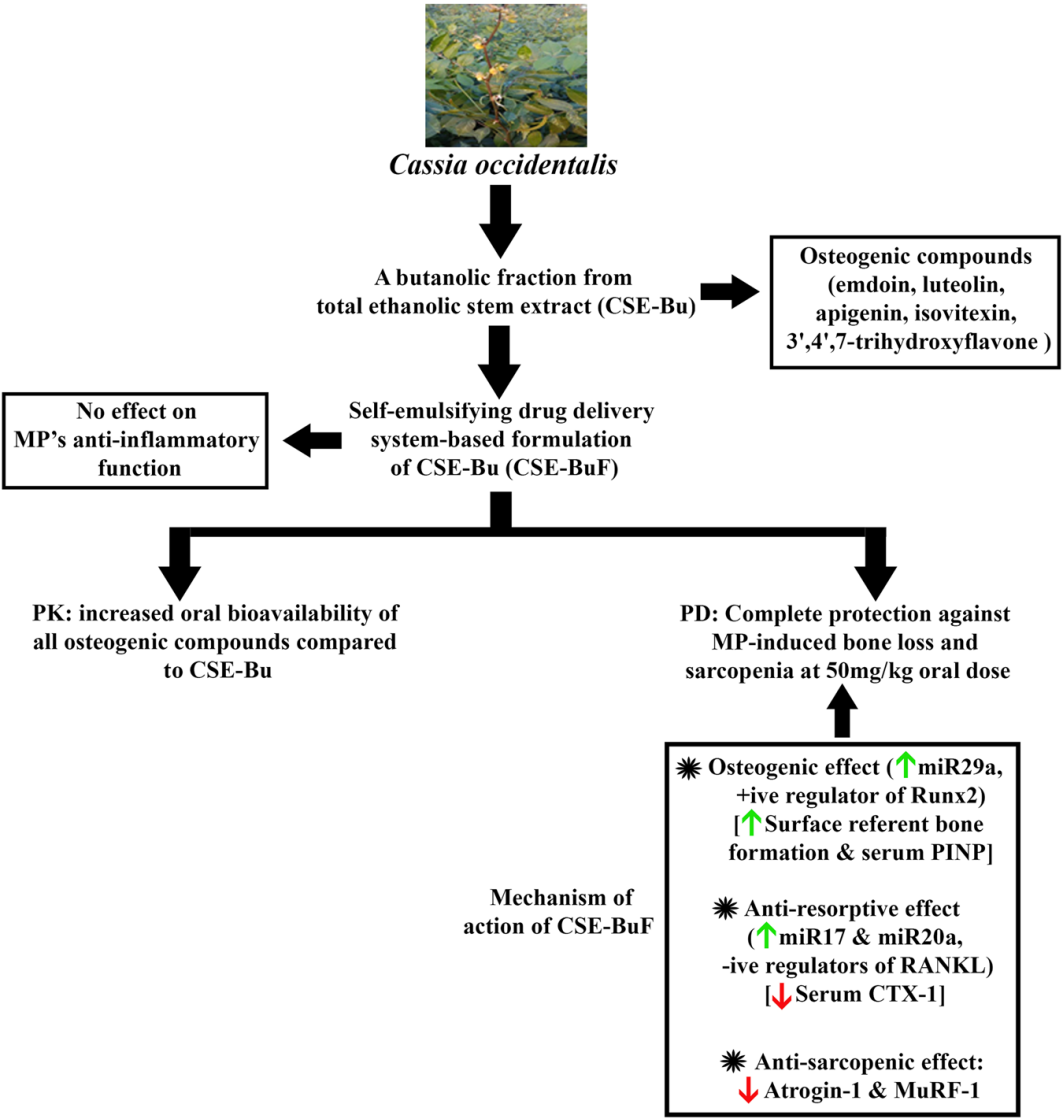
A schematic diagram illustrating the effcts of CSE-Bu or CSE-BUF on rat bones and skeletal muscle given concurrently with MP treatment. Picture used in this figure was taken by Subhashis Pal (author of this manuscript). PK, pharmacokinetics and PD: pharmacodynamics.

High doses of GCs are clinically used not only for RA but also for other chronic inflammatory conditions such as chronic obstructive pulmonary disease and inflammatory bowel disease. Therefore, impeding the anti-inflammatory effect of GCs on the way to protect bone is clinically unacceptable. Induction of GILZ by GCs is essential for their anti-inflammatory action and we observed that MP robustly up regulated GILZ and CSE-BuF did not block this effect in thymus and bone. Besides, CSE-BuF had no effect on the MP -mediated IL-1β suppression in serum. These data suggested that CSE-BuF could be prophylactically used for preventing MP-induced osteo-sarcopenia without hindering the anti-inflammatory effect of the drug. Figure 7 is a schematic representation of the major findings of this study.

From this preclinical study, we conclude that CSE-BuF is an effective pharmacotherapy for mitigating the osteo-sarcopenic impact of chronic GC treatment without hindering the anti-inflammatory action of GC, and thus should be further developed for clinical translation.

## Material and Methods

### Chemicals

Methylprednisolone (MP), calcein, hematoxylin, eosin, oleic acid, tocopherol succinate, ethylene glycol 1000 were purchased from Sigma-Aldrich (USA). 1,2 ethanediol was purchased from Merck Millipore (USA) and Tween80 was purchased from Himedia (India). PINP and CTX-1 ELISA kits were purchased from MyBioSource (USA). All primers were purchased from IDT (USA). MicroRNA assay system was purchased from Invitrogen (USA). Atrogin 1 antibody (ab168372) was purchased from Abcam (UK), MuRF 1 antibody (sc-32920) was purchased from Santa Cruz (USA) and GAPDH antibody (MAB374) was purchased from Merck Millipore (USA)^14^.

### Animals

All animal experimental procedures were prior approved (Institutional Animal Ethics Committee approval no. CDRI/IAEC/2014/145) and conducted as per the guidelines laid by the Committee for the Purpose of Control and Supervision of Experiments on Animals. All animals used for the study were obtained from National Laboratory Animal Center, CDRI and were subjected to 12 h dark–light cycle under controlled temperature (23–25 °C) and humidity (50–60%)^14^.

### Preparation of formulation

#### Self-emulsifying drug delivery system (SEDDS) development

The butanolic fraction from stems of *Cassia occidentalis* was prepared (CSE-Bu) as reported previously^14^. SEDDS were prepared by dispersing CSE-Bu (10% w/w) in accurately weighed quantities of oleic acid (55 to 70% w/w), surfactant (polysorbate 80), 20–30% w/w, co-surfactant (propylene glycol), 20–30% w/w and solubilizer (PEG 400) 5–10% w/w and were placed in a beaker and mixed thoroughly using magnetic stirrer to obtain a clear yellow solution. Extract containing formulation was prepared by dissolving 10 g extract in 90 g surfactant mix under sonication followed by magnetic stirring at 45 °C for 12 hrs. A transmittance of 100% was used to screen the composition to get the isotropic mixture. Based on globule size after 1:100 dilution with water the composition was optimized. The optimized composition of SEDDS was oleic acid (55% w/w), polysorbate 80 (20%w/w), propylene glycol (20% w/w) and PEG 400 (5%w/w). These SEDDS were stored at 25 °C. Blank SEDDS were prepared by varying the ratio of oil to surfactant to solubilizer to achieve transparent nanoemulsions after 1:100 dilution in Milli Q water^43^.

### Determination of globule size, size distribution, and Zeta potential

The globule size of formulations was determined by a Zetasizer Nano ZS model (Malvern Instruments) and the zeta potential was determined by laser Doppler anemometry using the Malvern Zetasizer. All the SEDDS were diluted with TDW to an appropriate concentration before determining the zeta potential. The measurements were carried out in the fully automatic mode. Each sample was analyzed thrice^43^.

### Pharmacokinetic studies

#### Liquid Chromatography-Tandem Mass spectrometric (LC-MS/MS) conditions

All analytes were analyzed using API-QTRAP 4000 mass spectrometer (ABSciex, Canada) equipped with electrospray ionization (ESI) source in negative ion ([M-H]^−^) mode [apigenin, isovitexin, emodin, luteolin, 3′,4′,7-trihydroxyflavone (THF), and fenofibric acid (FFA)] and quantified using Analyst 1.6 software (Applied Biosystems, MDS Sciex Toronto, Canada). Quantification was performed by multi-reaction monitoring (MRM), by monitoring the parent ion to product ion transition of 269.3/116.9 for apigenin, 431.2/311 for isovitexin, 269.1/224.9 for emodin, 284.9/133 for luteolin, 268.9/132.8 for THF, 316.9/230.9 for FFA (IS for apigenin, isovitexin, emodin, luteolin, and THF). Compound parameters, viz. declustering potential (V), entrance potential (V), collision energy, collision exit potential were −76, −10, −49, and −5 for apigenin; −100, −10, −20 and −15 for isovitexin; −100, −10, −40, and −10 for emodin; −100, −10, −50, and −20 for luteolin; −100, −10, −40, and −10 for THF; −55, −10, −12 and −10 for FFA; respectively. Source parameters, viz. curtain gas (psi), collision gas, ionspray voltage (V), ion source temperature (°C), nebulizer gas (GS1, psi) and turbo ion gas (GS2, psi) for apigenin, isovitexin, emodin, luteolin, THF, and FFA were 30, high, −4500, 500, 50, 50 respectively.

Shimadzu UFLC system (Kyoto, Japan) equipped with a binary pump (LC-20AD), a degasser (DGU-20A3), an autosampler (SIL-HTc) and a column oven (CTO-20AC) was used to inject the samples. The chromatographic separation of apigenin, isovitexin, emodin, luteolin, THF and FFA were performed on Phenomenex Luna (C18 (2), 100A, 150 × 4.6 mm, 3 μ) column with a mobile phase consisting of methanol: 10 mM ammonium acetate, 95:05 (v/v) at 0.6 mL/min flow rate. The temperature of the column oven was set at 40 °C during analysis. 10 µL samples were injected for quantification^44^.

### Extraction method

For extraction of apigenin, isovitexin, emodin, luteolin, THF, liquid-liquid extraction (LLE) method was applied for processing rat plasma. Plasma (100 µL) was incubated with 50 µL β-glucuronidase enzyme (2 mg/mL in 100 mM ammonium acetate buffer pH 5), and 50 µL ammonium acetate buffer (100 mM, pH 5) for 2 h at 37°C on water shaking bath. 200 µL ACN containing IS (FFA, 100 ng/mL) was added to plasma, vortex for 15 sec, and then 100 µL 0.1% formic acid (in 10 mM ammonium formate) was added. After vortexing for 5 min on vortexer (IKA^(R)^ VIBRAX VXR basic) at 1200 rpm, 2 mL ethyl acetate was added. The mixture was again vortexed for 10 min and then centrifuged at 10000 rpm for 5 min. The supernatant (1.5 mL) was transferred into Ria vial and completely dried in a TurboVap. The dried residue was reconstituted with 100 µL of mobile phase and analyzed by LC-MS/MS^44^.

### Determination of plasma levels of osteogenic compounds

For determination of osteogenic compounds in the plasma of rats given CSE-Bu and CSE-BuF, we recruited male Sprague Dawley (SD) rats (220 ± 20 g) and were fasted overnight with free access to water. Rats were divided into 2 groups consisting of 6/group. One group received CSE-Bu (250 mg/kg/oral) and the other received CSE-BuF (250 mg/kg/oral). Blood (300 µL) was collected into 0.5 ml centrifuged tube containing EDTA at 0.25, 0.5, 1, 2, 4, 8, 12, 24, and 48 h and plasma was separated followed by centrifugation at 10000 rpm for 10 min and stored at -80 °C for further analysis. For pharmacokinetic analysis, plasma concentration versus time was plotted and analyzed by non-compartmental analysis method using WinNonlin (Pharsight, Mountain View, CA) software.

### *In vivo* studies

#### Femur osteotomy model

Adult male SD rats (200 ± 20 g) were taken for the study and divided into different groups. Femur osteotomy was done according to our previously published method^14^. Thirty-six rats were randomly divided into 6 equal groups, vehicle (water), vehicle formulation (blank SEDDS without extract), CSE-Bu at 50- and 100 mg/kg and CSE-BuF at 50- and 100 mg/kg. All treatments were given by oral route daily for 12 days. 24 h before sacrifice all animals received an intraperitoneal injection of calcein (20 mg/kg). Bone volume in the callus was assessed by SkyScan 1076 µCT scanner (SkyScan, Belgium). For calcein binding study, bones were embedded in an acrylic material and sectioned (50 µm) using Isomet Bone cutter (Agra, UP, India). Photographs were taken under a confocal microscope (Carl Zeiss, Germany). Sections were viewed and intensity of calcein binding was calculated using Carl Zeiss AIM 4.2 image analysis software^14,45^.

#### Glucocorticoid-induced osteoporosis (GIO) model

Thirty male SD rats (260 ± 20 g) were randomly divided into 3 equal groups: vehicle (blank SEDDS without extract), MP, and MP + CSE-BuF. MP was administered daily by subcutaneous route (5 mg/kg in 50 µl). CSE-BuF was administered daily by oral gavage (50 mg/kg in 1000 µl). All treatments lasted for 4 weeks. There was no mortality in any group throughout the treatment duration. For dynamic histomorphometry study, each animal received two subcutaneous injections of calcein at 10 days interval before sacrifice. At the end of treatment, animals were killed, bones were collected and fixed for different experiments described below^46^. Bones were fixed in 70% isopropanol for histomorphometry analysis and µCT scanning, for biomechanical testing bones were preserved at −20 °C^14^.

#### Body composition analysis

Body composition of live rats was analyzed using an Echo-MRI Body Composition Analyzer E26-226-RM (EchoMRI LLC, USA) as per our previously described method^14,47–50^.

#### Micro CT (μCT) analysis

Micro-computed tomographic (μCT) analyses of bones were carried out using Sky Scan 1076 μCT scanner (SkyScan, Belgium). Scanning was done at 70 kV, 142 mA using a 1 mm aluminum filter at a resolution of 18 μm/pixel. Cross sectional reconstruction was made using Nrecon software based on modified feldkamp algorithm. To analyze trabecular region, 100 slices region of interest (ROI) was drawn below the growth plate. For cortical bone analysis 100 consecutive image slides were selected at mid diaphysis region. CTAn software were used for micro architectural parameter measurement. Bone mineral density (BMD) was measured by using hydroxyapatite phantom rods of 4 mm diameter with known BMD (0.25 g/cm^3^ and 0.75 g/cm^3^) as calibrator^14,51^.

#### Bone strength measurement

Bone mechanical strength was measured by three-point bending test for femur diaphysis and compression test for L5 vertebrae using a bone strength tester TK 252 C (Muromachi Kikai Co. Ltd, Tokyo, Japan) according to a previously published protocol^14,52^.

#### Bone dynamic histomorphometry

For dynamic histomorphometric measurements, double calcein labeling was performed with an interval of 10 days between two calcein injections. We measured the periosteal perimeter, single-labeled surface (sLS), double labeled surface (dLS), and interlabeled thickness (IrLTh). These data were used to calculate mineralizing surface/bone surface (MS/BS), mineral apposition rate (MAR, and bone formation rate (BFR) as follows: MS/BS = (1/2 sLS + dLS)/BS (%); MAR = IrLTh/10 days (μm/day); BFR/BS = MAR × MS/BS (μm^3^/μm^2^/day)^14,53^.

#### Serum biochemical marker measurement

The serum samples were collected from blood by centrifugation at 3000 rpm. Serum cross-linked C-telopeptide of Type I collagen (CTX-I), procollagen type I N-terminal propeptide (PINP), sclerostin (SOST), interleukin 1 beta (IL-1β) levels were determined by ELISA (MyBioSource, USA) following the manufacturer’s protocols^14^.

#### Determination of osteoblast- and osteoclast-specific genes and miRNAs

Trizol extraction method was used for total RNA extraction from proximal femurs (devoid of bone marrow) using TRIzol reagent following manufacturer protocol (Cat # 15596026, Invitrogen, USA). SYBR Green chemistry was used to perform quantitative determination of receptor activator of nuclear factor kappa-Β ligand (RANKL), osteoprotegerin (OPG), tartrate-resistant acid phosphatase (TRAP), osteocalcin (OCN), dentin matrix protein 1 (DMP-1), matrix extracellular phosphoglycoprotein (MEPE) and the housekeeping gene glyceraldehyde-3-phosphate dehydrogenase (GAPDH) expression from bones of different treatment groups. Glucocorticoid-induced leucine zipper (GILZ) expression was measured in thymus and proximal femurs devoid of bone marrow. Primers were designed based on published sequence using the Universal ProbeLibrary (Roche Applied Sciences, USA). Primer sequences are listed below:

**RANKL**: Forward 5′-AGACACAGAAGCACTACCTGA-3′, Reverse 5′-GGCCCCACAATGTGTTGTA-3′; **OPG**: Forward 5′-GGAGCTCGAATTCTGCTTGA-3′, Reverse 5′-GAAGAACCCATCCGGACATC-3′; **TRAP**: Forward 5′-CAGGGACGGGAGAGATTGG-3′, Reverse 5′-GCTGTACAGTGAGCCAGGA-3′; **OCN**: Forward5′-CACCGTTTAGGGCATGTGTT-3′, Reverse 5′-TCCTGGAGAGTAGCCAAAGC-3′; **DMP-1**: Forward 5′-CAGAGGTTCCACACGTAGCA-3′, Reverse 5′-TCAATGGAGAAAGCCTATGTGA-3′; **MEPE**: Forward 5′-AGAAGCCAAGCTTCCCTGA-3′, Reverse 5′-ACAGCCTGCATCGTCACAC-3′; **GILZ**: Forward 5′-GGGATGTGGTTTCCGTTAAA-3′, Reverse 5′-TTGTTGTCTAGGGCCACCA-3′;**GAPDH**: Forward 5′-TGGGAAGCTGGTCATCAAC-3′, Reverse 5′-GCATCACCCCATTTGATGTT-3′.

cDNA was synthesized using Revert Aid cDNA Synthesis Kit (Fermentas, USA) using 2 μg total RNA. All genes were analyzed using Light Cycler 480 real-time PCR machine (Roche Molecular Biochemicals, USA).

TaqMan assay (Thermo Fisher Scientific, USA) was used to determine miRNA expression in bones. Specific assay was designed based on published sequence using Thermo Fisher miRNA assay design tool. Assay ID of different miRNA was; hsa-miR-29a (OO2112), hsa-miR-17 (OO2308), hsa-miR-20a (OOO580) and U6 snRNA (OO1973)^54^.

#### Evaluation of the anti-inflammatory response

After 4 weeks of treatments with vehicle, MP (5 mg/kg, subcutaneous), and MP + CSE-BuF (50 mg/kg, oral) thymus and proximal femurs (devoid of bone marrow) were collected from all rats to measure GILZ mRNA levels. SYBR Green chemistry was used to perform quantitative determination of GILZ, a mediator for the glucocorticoid-mediated anti-inflammatory response^55^. Serum IL-1β level were measured to evaluate the effect of CSE-BuF on glucocorticoid-mediated anti-inflammatory response.

#### Muscle histomorphometry

Gastrocnemius muscles were carefully dissected and stored in chilled PBS for 48 h. Tissues were then transferred to 30% sucrose solution (in PBS) at 4 °C until they sank to the bottom. Tissues were embedded in OCT tissue freezing medium (Leica Bio systems, India) and cryo-sections (7 µm) were made using a Cryotome™ FSE Cryostat (Thermo Scientific). Tissue sections were collected on poly-L-lysine coated slides. For H&E staining, sections were rehydrated in PBS for 10 min and fixed in 10% formalin for 3 min. Then H&E staining was performed and mounted with DPX (Sigma). Quantitative analysis for determining cross-sectional area and Feret’s diameter of the muscle fibers were performed using Image J software^50,56^.

#### Immunoblotting

Gastrocnemius muscle samples were collected from each group and protein lysates were prepared to determine the levels of muscle atrogenes [atrogin 1], and muscle ring finger protein (MuRF1). Relative expression of these proteins was detected by immunoblot analysis using specific antibodies againts atrogin 1 (Abcam; 1:1000 dilution), MuRF1 (Santa Cruz Biotechnology; 1:1000 dilution), and GAPDH (Merck Millipore; 1:1000 dilution). Densitometric analyses of immunoblots were performed using ImageJ software^50^.

#### Statistics

Data are expressed as mean ± SEM unless otherwise indicated. The data obtained were subjected to one-way ANOVA followed by post hoc Dunnett’s multiple comparison tests of significance for *in vivo* studies using GraphPad prism 5^14^.

## Supplementary information

Supplementary Information.

## Data Availability

The datasets during and/or analyzed during the current study available from the corresponding author on reasonable request.
