# Supplementary material for: A butanolic fraction from the standardized stem extract of Cassia occidentalis L delivered by a self-emulsifying drug delivery system protects rats from glucocorticoid-induced osteopenia and muscle atrophy
Source: Sci Rep. 2020 Jan 13;10:195. doi: 10.1038/s41598-019-56853-6 (PMC6957531; doi:10.1038/s41598-019-56853-6)

**A butanolic fraction from the standardized stem extract of *Cassia occidentalis* L delivered by a self-emulsifying drug delivery system protects rats from glucocorticoid-induced osteopenia and muscle atrophy**

Subhashis Pal^1^, Naresh Mittapelly^2^, Athar Husain^3^, Sapana Kushwaha^4^, Sourav Chattopadhyay^4,6^, Padam Kumar^5^, Eppalapally Ramakrishna^5^, Sudhir Kumar^5^, Rakesh Maurya^5^, Sabyasachi Sanyal^4,6^, Jiaur R. Gayen^3^, Prabhat R. Mishra^2^, Naibedya Chattopadhyay^1,*^

^1^Division of Endocrinology and Center for Research in Anabolic Skeletal Target in Health and Illness (ASTHI), CSIR-Central Drug Research Institute, Council of Scientific and Industrial Research, Lucknow 226031, India

^2^Division of Pharmaceutics, CSIR-CDRI, Lucknow 226031, India

^3^Division of Pharmacokinetics, CSIR-CDRI, Lucknow 226031, India

^4^Division of Biochemistry, CSIR-CDRI, Lucknow 226031, India

^5^Division of Medicinal & Process Chemistry, CSIR-CDRI, Lucknow 226031, India

^6^AcSIR, CSIR-Central Drug Research Institute Campus, Lucknow 226031, India

***Correspondence:** Naibedya Chattopadhyay, Ph.D., Endocrinology Division, CSIR-Central Drug Research Institute, Lucknow 226031, India. Tel: +91-522 2613894; Fax: +91-522 2623938; Email: [n_chattopadhyay@cdri.res.in](mailto:n_chattopadhyay@cdri.res.in).

**Keywords**: Folk medicine; Osteoanabolic; Osteogenic formulation, Anti-resorptive; Fracture healing; Glucocorticoid-induced osteoporosis; Bone strength.


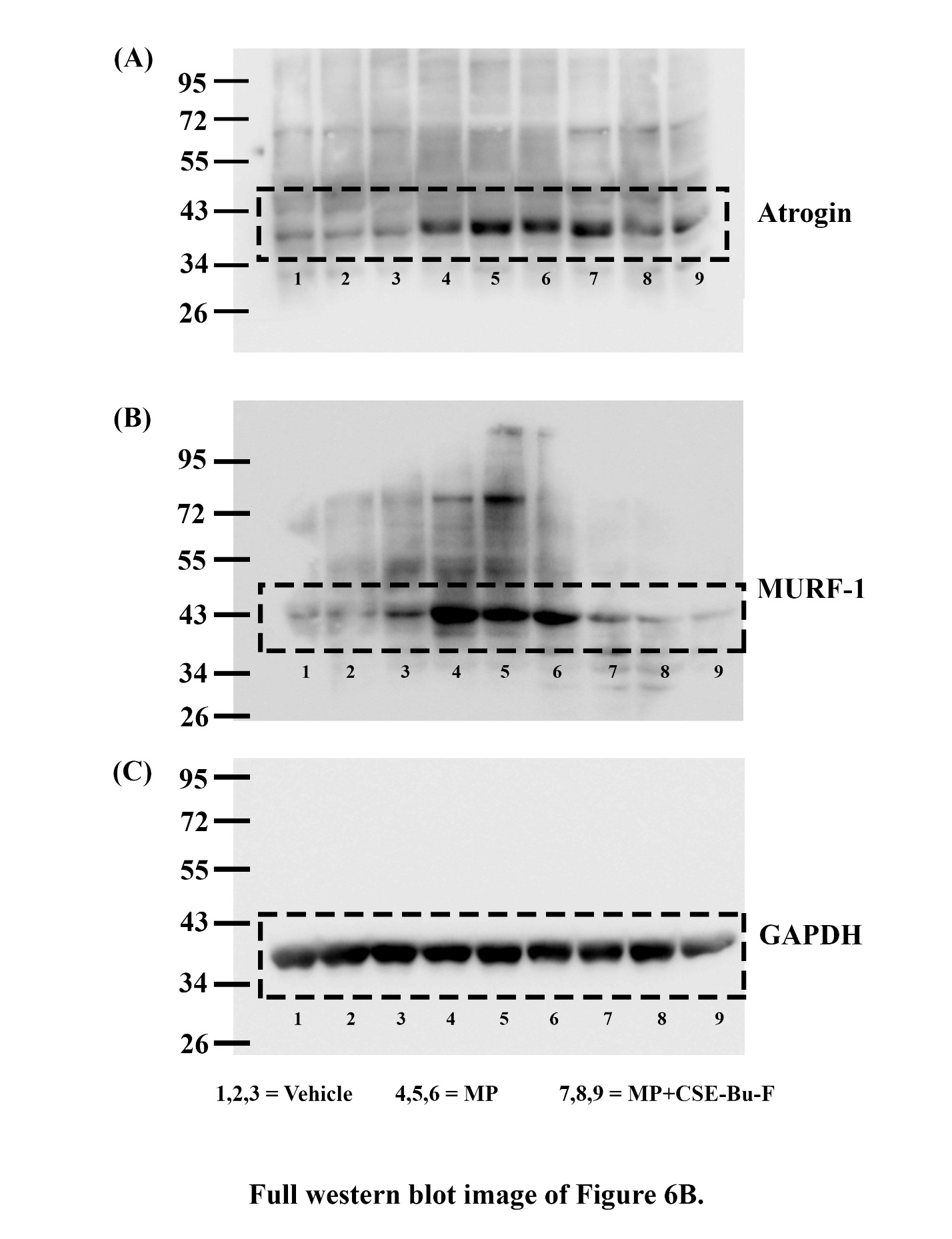

Supplement: Supplementary file 1 — Supplementary Information. [file 41598_2019_56853_MOESM1_ESM.docx]
